# Different road maps for ventricular tachycardia ablation

**DOI:** 10.1007/s12471-020-01507-w

**Published:** 2020-10-20

**Authors:** L. R. A. Olde Nordkamp, S. M. Boekholdt, J. R. de Groot

**Affiliations:** Department of Cardiology, Heart Center, Amsterdam UMC, University of Amsterdam, Amsterdam Cardiovascular Sciences, Amsterdam, The Netherlands

Catheter ablation is being used increasingly to treat patients with ischaemic scars who are suffering from recurrent haemodynamically tolerated and untolerated ventricular tachycardia (VT). Its main goal is to locate and ablate isthmuses critical for sustaining the VT. Functionally, this is achieved by using pacing manoeuvres and entrainment mapping to identify which areas represent critical parts of the reentrant circuit [1]. Alternatively, the substrate of the VT is assessed on a structural level by identifying low-voltage or fractionated local electrograms, without demonstrating that these abnormalities contribute to the arrhythmia. A randomised trial comparing substrate-based VT ablation with functional VT ablation using pacing manoeuvres and entrainment showed that substrate modification causes lower rates of VT recurrence and hospitalisation than functional VT ablation as well as a trend toward lower mortality [2].

Currently, in most cases, extensive electro-anatomical mapping is used to delineate bipolar and unipolar low-voltage areas, and to identify fragmented electrograms and late potentials. However, electro-anatomical mapping can be a time-consuming process, and may be hampered by inaccurate delineation of intramural scars or attenuation of electrograms by epicardial fat. Therefore, non-invasive imaging using computed tomography (CT) or cardiac magnetic resonance imaging (CMR) may help to delineate the arrhythmogenic substrate accurately, and subsequently guide substrate-based ablation of VTs. Whether this approach results in higher efficacy of the procedure or better outcomes is unclear so far. In this issue of the *Netherlands Heart Journal*, Hendriks et al. present a meta-analysis of 38 studies including a total of 7748 patients with ischaemic VT undergoing VT ablation, of whom 224 patients underwent VT ablation guided by pre-procedural imaging [3]. Hendriks et al. conclude that VT-free and overall survival in patients with ischaemic VT is higher with image-guided VT ablation than with conventional VT ablation (82% vs 59% for VT-free survival and 94% vs 82% for overall survival, respectively). They suggest that visualising myocardial scar, in these studies done by echocardiography, CT, single-photon emission computed tomography or CMR, may facilitate substrate-guided ablation procedures and consequently may improve long-term outcome [3].

There are several potential advantages of making use of non-invasive imaging to guide the operator to the optimal ablation location. Imaging before or integrated during VT ablation allows three-dimensional delineation of the substrate. With electro-anatomical mapping alone, defining the complex three-dimensional geometry of a scar is difficult, both because of its two-dimensional nature and because of its limited spatial resolution. Detailed imaging before or during a VT ablation can facilitate identification of the epicardial border zone of the scar, which is involved in the majority of VTs [4]. Also, particularly during epicardial procedures, ablation in the proximity of coronary arteries is challenging, and it may be difficult to differentiate epicardial fat from the myocardium. Image-guided VT ablation can potentially overcome these pitfalls. Finally, accurate imaging of the ischaemic scar may also be relevant for cardiac radio-ablation [5], whereby the substrate is ablated with radiation.

However, some limitations of image-guided VT ablation apply. Due to its potential for tissue characterisation, CMR has important advantages over other modalities such as echocardiography and CT, which are mainly used to delineate areas of wall thinning. However, a large proportion of VT patients carry an implantable cardioverter defibrillator (ICD), meaning potential safety concerns for both the patient, including tissue heating adjacent to lead electrodes, and the device, although the absolute risks appear to be low. ICDs can provoke artefacts that hamper the accuracy of substrate delineation. Novel sequences such as wideband late gadolinium enhancement (LGE) have been introduced for patients with cardiac devices, but current applicability is limited by its resolution, which is unlikely to be sufficient for defining the detailed myocardial architecture of the arrhythmogenic substrate [6]. Novel CMR sequences are being developed for high-resolution myocardial imaging in patients with cardiac devices [7–9], but these techniques have not been studied extensively to date, particularly not for VT ablation purposes. Given the device-related issues with CMR, many centres perform CMR routinely before ICD implantation. Such a protocol leads to the availability of CMR data on these ICD patients once they develop VT, but this strategy is susceptible to changes in volume, orientation and heart rhythm, which can result in discrepancies in registering scar data from CMR to real-time electro-anatomical mapping [6]. Real-time CMR to guide ablation would help to overcome these limitations, but at the moment CMR-based ablation is an experimental technique, only available in very few centres in the world.

The meta-analysis by Hendriks et al. sheds light on an interesting paradigm change in the approach to the ischaemic scar during VT ablation. However, due to the nature of the available evidence, this was an observational comparison of individual studies without a predefined definition of image guiding. Studies were presumed to represent image guided VT ablation when imaging directly contributed to the ablation procedure. Hence, when pre-procedural imaging was performed but did not directly affect the ablation workflow, studies were stratified as conventional ablation. There were important differences in the clinical and electrophysiological characteristics between studies employing an image guided approach and those using conventional ablation. Patients undergoing image-guided ablation tended to be younger (64 years vs 66 years), had a higher left ventricular ejection fraction (34% vs 32%), had more previous VT ablations (32% vs 15%), had an ICD less often (55% vs 94%) and were more often subjected to an epicardial ablation approach (37% vs 6%). Each of these factors is expected to impact on VT-free survival and mortality. Moreover, there was considerable overlap in patients in the image-guided VT ablation group, since four out of five studies (two from Barcelona, two from Bordeaux) were from the same cardiology centre and CMR data were available in only a subset of patients, with the remainder undergoing CT or echocardiography. For further determination as to whether image-guided VT ablation can be considered a superior approach compared to conventional VT ablation, a more stringent, randomised comparison of different strategies is needed [10]. Nevertheless, the authors should be commended for their informative and hypothesis-generating efforts to increase our understanding of strategies aimed at improving VT ablation therapies.

## References

[1] Stevenson WG, Friedman PL, Sager PT, Saxon LA, Kocovic D, Harada T (1997). Exploring postinfarction reentrant ventricular tachycardia with entrainment mapping. J Am Coll Cardiol.

[2] Di Biase L, Burkhardt JD, Lakkireddy D, Carbucicchio C, Mohanty S, Mohanty P (2015). Ablation of stable VTs versus substrate ablation in ischemic cardiomyopathy: the VISTA randomized multicenter trial. J Am Coll Cardiol.

[3] Hendriks AA, Kis Z, Glisic M, Bramer WM, Szili-Torok T (2020). Pre-procedural image-guided versus non-image-guided ventricular tachycardia ablation—a review. Neth Heart J.

[4] Fernández-Armenta J, Berruezo A, Andreu D, Camara O, Silva E, Serra L (2013). Three-dimensional architecture of scar and conducting channels based on high resolution ce-CMR: insights for ventricular tachycardia ablation. Circ Arrhythm Electrophysiol.

[5] van der Ree MH, Blanck O, Limpens J, Lee CH, Balgobind BV, Dieleman EMT (2020). Cardiac radioablation—a systematic review. Heart Rhythm.

[6] Mukherjee RK, Whitaker J, Williams SE, Razavi R, O’Neill MD (2018). Magnetic resonance imaging guidance for the optimization of ventricular tachycardia ablation. Europace.

[7] Basha TA, Akçakaya M, Liew C, Tsao CW, Delling FN, Addae G (2017). Clinical performance of high-resolution late gadolinium enhancement imaging with compressed sensing. J Magn Reson Imaging.

[8] Shin T, Lustig M, Nishimura DG, Hu BS (2014). Rapid single-breath-hold 3D late gadolinium enhancement cardiac MRI using a stack-of-spirals acquisition. J Magn Reson Imaging.

[9] Dzyubachyk O, Tao Q, Poot DH, Lamb HJ, Zeppenfeld K, Lelieveldt BP (2015). Super-resolution reconstruction of late gadolinium-enhanced MRI for improved myocardial scar assessment. J Magn Reson Imaging.

[10] de Groot JR (2019). What if there is no prospective, double blind, randomised trial?. Neth Heart J.

